# Cholera in Egypt

**Published:** 1902-08-02

**Authors:** 


### Cholera in Egypt.

The outbreak of cholera in Egypt shows again how futile all, even the best devised, schemes of quarantine are apt to prove, when behind the *cordon* lies a people whose habits and surroundings afford no protection against the disease in question. Cholera in its epidemic form is essentially a water-borne disease, the spread of which depends upon dirty social habits and unprotected water-supplies; while Egypt is a country in which not only are the habits of the people filthy to a degree, but in which the water-supply is peculiarly open to pollution. The one source from which Egypt draws its water is the Nile, which in turn is the one sewer into which all the country drains.

Far away to the south, in the neighbourhood of of Khartoum, the Nile, issuing from the fertile Soudan, starts upon its solitary course to the Mediterranean. For more than a thousand miles, without receiving a single tributary, it flows through a country which, but for its waters, would be a desert, and which, in many pla ces, is in fact a desert up to its very banks. Throughout the whole length of Egypt all the water used for every purpose comes primarily from the river, and back to the river flows all the excess beyond what is evaporated. That being so it is easy to understand what a serious matter it is to Egypt to be attacked by a water-borne disease like cholera.

According to Egyptian tradition the rising Nile washes away cholera and every other evil, but certainly that has not been the case in recent epidemics. At some seasons, as at the present moment, Egypt is a land of flowing water; at other times when the Nile is low it is a land of stagnant canals, and although in Cairo and Alexandria water is, in a manner, "laid on," the source even of such water is by no means above suspicion, and a large proportion of natives in Cairo use water drawn direct from the river and canals. The habits and customs of the people, again, render it a very difficult task to control the spread of the disease. As in the epidemic of 1883, when according to official figures 58,000 persons, and probably in reality not less than 80,000 persons, perished by the disease, so also in that of 1895–1896, when the loss of life amounted to between 18,000 and 19,000, every effort of the sanitary authorities was opposed by the people, this opposition in some cases being carried to such an extent that force had to be employed. Nor was this resistance confined entirely to the more ignorant classes, as witness the riots at the El Azhar university and mosque when several persons were killed. As it was, so it will be. Egypt does not change in a day, so that we must not be too optimistic in our forecast. The difficulty always has been to keep the water-supplies reasonably pure, and this arises from the habits of the people, habits which are ingrained and cannot be eradicated by a few years of education. Dr. Bruce Low, in a report on the subject to the medical officer of the Local Government Board, says: "The water-supplies of Egypt are largely capable of pollution, and are, in fact, largely polluted. None of the towns or villages of Egypt has any satisfactory system of drainage. The habits of the people are filthy. The ablutions prescribed by religion are commonly performed at the nearest water-supply. The bank of the river or canal is generally utilised as a public latrine, and the condition of the foreshore is inexpressibly filthy. Washing of soiled clothing, the performance of ablutions prescribed by religion after the calls of nature, and the drawing of drinking water, may all be seen in the act of being carried on within a few yards of one another." In a country so peculiarly placed in regard to its water-supply, and with a people in whom such habits are in ingrained as a second nature, it is not to be wondered at that cholera is difficult of control; nor need we be surprised that the attempt which has been made this year to prevent infection from Mecca entering Egypt should have failed.

At El Tor a vast and carefully designed quarantine station has been established. But there are other roads to Egypt besides El Tor. Big steamers can be inspected and disinfected, but coasting traffic and small boats crossing the Red Sea cannot easily be brought under control. Moreover, the investigations of Koch and others, which showed that after recovery from an attack of cholera a man's dejecta may for some time contain cholera bacilli, throw doubt upon the trustworthiness of quarantine even when most perfectly carried out.

A country's protection from cholera depends upon its possession of a system of water-supply which is capable of being protected from pollution, and it is the absence of such a system in Egypt that leaves that country at the mercy of a single imported case. Nor is Egypt alone in this, and although things are far better now than they were a few years ago, this Egyptian outbreak must of necessity give rise to the most extreme anxiety along the whole of the Mediterranean littoral.

